# Assessing the Economic Burden of Disease in Migrants: The Diabetes Case in Northern Italy

**DOI:** 10.3390/ijerph17072250

**Published:** 2020-03-27

**Authors:** Pietro Ferrara

**Affiliations:** Research Centre on Public Health, University of Milan—Bicocca, 20900 Monza, Italy; p_ferrara@alice.it

**Keywords:** cost of care, economic burden of disease, migrant health, minority health, refugees health

## Abstract

By considering the prevalence of diabetes in migrants living in northern Italy, this editorial aims to highlight the need for the measurement of the economic burden of disease in migrant and refugee populations. This type of assessment is potentially useful to address the emerging challenges of the migrant health burden, by enhancing the ability of health systems and policies to respond to migrants’ health needs—in terms of health care and promotion—leading to significant better conditions for future multicultural environments.

## Foreword

In the last decades, progress has been made in research on migrant and refugee health, mainly in terms of epidemiological investigations and health determinant identification. However, little or nothing has been investigated into the measurement of the economic burden of disease in the host country population. Defining health costs is a priority of public health research; costs and benefits analyses are among the main tools of health policymaking. Indeed, knowing and recognizing the real impacts of the care of migrants and refugees on healthcare systems is crucial information in view of continuous improvement of a smoother and faster reallocation of health resources.

According to 2019 estimates, migration to Italy accounts for around 6.2 million people, constituting 10.3% of the total Italian population, variously distributed in the country [1,2]. In this framework, the aim of the present report is to focus attention on the economic burden of disease in migrants, based on estimations of cost of care for diabetes across immigrant groups, using disease prevalence in northern Italy, the area with the highest number of migrants [1]. In this area, Fedeli and colleagues found that among immigrant groups aged from 20 to 59 years, the estimable diabetes prevalence rate was around 4.4%, being 2.9-fold higher than that of same-age Italians [3], with estimable 15,534 sick subjects.

Among non-communicable chronic diseases, diabetes is recognized to have a major impact on country-level healthcare expenditures in terms of hospitalizations and ongoing care (drugs, outpatient care), as well as other indirect and intangible costs [4]. In Italy, it is estimated that the disease annually burdens the National Healthcare Service (NHS) with €9 billion of direct costs, with a predictable rising trend [4]. The average annual cost of care for a single diabetic patient totals €2756 [4].

Based on these numbers, the care of these 15,534 diabetic migrants requires a cumulative expenditure of €42.8 million per year. Beyond this baseline cost, diabetes also impacts the use of healthcare resources, with high costs due to complications, which can reach €17,500 per patient/year in case of major amputation due to peripheral artery disease [5].

This cursory analysis underlines the economic impact of migrants’ chronic conditions on health costs, taking into account only one index disease in one region—and excluding elderly immigrant populations (due to limitations in data [3]). First, we want to call attention to the importance of including this type of economic analysis at the country-level and in cross-national studies of healthcare costs. Second, with this communication, we hope to provide a starting point for a more accurate evaluation of the economic burden of disease in migrants.

As known, understanding the economic impacts of ill-health is crucial for the sustainability of healthcare systems—and to respond to policy questions on the social consequences of disease and disability. Migrant and refugee policies vary substantially among developed countries, and increasing attention has been placed on their health during the last decade. However, the economic aspects of health and social care of this population fades into the background, while appropriate research should focus on that which also considers the ability of hosting countries to maintain economic growth.

In conclusion, the idea is to point out that these analyses are potentially useful to address the emerging challenges of the migrant health burden, by enhancing the ability of health systems and policies to respond to migrants’ health needs—in terms of health care and promotion—leading to significant better conditions for future multicultural countries.
